# Age of diagnosis, service access, and rights of autistic individuals in Argentina: Caregivers reports of changes and similarities across time

**DOI:** 10.3389/fpsyt.2022.915380

**Published:** 2022-08-16

**Authors:** Maria Cecilia Montenegro, Estefani Bernal, Sebastian Cukier, Daniel Valdez, Alexia Rattazzi, Gabriela Garrido, Analia Rosoli, Cristiane Silvestre Paula, Ricardo Garcia, Cecilia Montiel-Nava

**Affiliations:** ^1^Department of Psychological Sciences, University of Texas Rio Grande Valley, Edinburg, TX, United States; ^2^Research Department, Programa Argentino para Niños, Adolescentes y Adultos con Condiciones del Espectro Autista (PANAACEA), Buenos Aires, Argentina; ^3^Research Department, Facultad Latinoamericana de Ciencias Sociales (FLACSO), Buenos Aires, Argentina; ^4^Department of Child and Adolescent Psychiatry, Universidad de la República, Montevideo, Uruguay; ^5^Research Department, Organización Estados Iberoamericanos para la Educación, la Ciencia y la Cultura (OEI), Santo Domingo, Dominican Republic; ^6^Research Department, Programa de Pós-Graduação em Distúrbios do Desenvolvimento Universidade Presbiteriana Mackenzie (UPM), São Paulo, SP, Brazil; ^7^Research Department, Universidad de Chile, Santiago, Chile

**Keywords:** caregivers, ASD, Argentina, age of diagnosis, first concern, service barriers, priorities

## Abstract

**Background:**

Many countries have developed health initiatives to protect those with disabilities and developmental concerns in the past few years. However, the needs of autistic individuals are still short of being fulfilled. Partially due to limited research expenditure, which would allow bridging the gap between evidence and practice, the long time it takes to implement passed laws, and the limited operationalization of inclusive policies.

**Objective:**

To quantitatively examine changes in the child's age at the time of caregiver's first developmental concerns and age of diagnosis of their autistic child across 5 years. Also, to address challenges experienced by caregivers (e.g., reported service barriers) and the work still needing to be done in Argentina based on caregivers' reports of their priorities (e.g., ensuring their child receives better services).

**Methods:**

Two independent samples of caregivers of autistic individuals were surveyed by the Red Espectro Autista Latinoamerica (REAL) in 2015 (*n* = 763) and the World Health Organization (WHO) in 2020 (*n* = 422). Similar items in both surveys were compared through descriptive inferential analysis and chi-square tests for categorical variables.

**Results:**

Compared to the 2015 sample, for the 2020 sample, more caregivers reported an earlier age of first concerns and an earlier age of a professional diagnosis. In the 2020 sample, more children diagnosed before the age of three had a doctor or a teacher noticing the first developmental concern. Also, in this sample, fewer caregivers reported service barriers (e.g., limited availability, waitlist, costs, etc.) and a need for better social support and better health services. However, rates of caregivers indicating a need for more rights for autistic individuals and greater protection of existing rights increased. There was no change in the reported rate of family members who stopped working to care for the autistic individual. For both samples, there was statistically significant differences in individual (physician, teacher, caregiver) noticing first developmental concern and the age of diagnosis, with the majority having a caregiver noticing the first concern.

**Conclusion:**

The 5 years that separate both samples show an improvement in developmental concerns being noticed, a decrease in age of diagnosis, and an improvement in several service areas such as community awareness. Also, caregivers reported fewer barriers to service accessibility, thus suggesting a positive impact stemming from changes in public policies, non-profit organizations' work through awareness campaigns, and advocates' strives toward greater awareness. Nonetheless, a similar proportion of family members reported ceasing working to care for autistic individuals and perceived that the fundamental rights of their autistic children needed to be protected. These results imply that despite better care pathways in Argentina, there are still gaps when attempting to meet the needs of autistic individuals and their families. The present study provides a meaningful understanding of existing gaps and help exemplify the perceived improvements when non-profit agencies and advocates promote increased rights and community awareness in addition to the established laws focusing on ASD.

## Introduction

The global prevalence of Autism Spectrum Disorder (ASD) is estimated to be 1 in 100 (1) and is in stark contrast to estimates in the United States showing an increased prevalence of ASD to 1 in 44 (2). Low global prevalence estimates suggest barriers to identification in other parts of the world. Although countries like the United States have minimized barriers to accessing appropriate evaluation and intervention services for children with ASD, other countries have historically lagged in this regard due to socioeconomic and cultural factors that further deepen the treatment gap and limit ASD community awareness (3–6). One of these countries, Argentina, is plagued by economic disparity throughout its regions (7), and information regarding Autism prevalence is limited. One study found the prevalence rate of disability in children to be 3.2%, with 40% of these children being identified with ASD (8). This may be an underestimation, as this study only included children who had obtained a Unique Certificate of Disability (UCD), and it has historically been identified that a significantly larger proportion of children with disability do not apply for a UCD (9). Despite its status as an upper-middle income country, Argentina experiences socioeconomic, political, and environmental inequities, all factors that have been shown to contribute to health disparities (10). The most recent economic data indicates that 40.6% of the population of Argentina lives in poverty, with an additional 10.7% living in extreme poverty (7). Although Argentina's health care system is on the path to being one of universal health coverage, discrepancies in the access to and the provision of health services exist among the population. Most recent financial information indicates 36% of the country's population has no insurance and relies on the public health sector for health treatment (7). Moreover, due to the structural framework and fragmented funding of the healthcare system, disparities in screening, time to diagnosis, and treatment of serious conditions have been found to vary among lower and higher-income districts and regions (11). These disparities could have a significant impact on the identification and treatment of conditions such as ASD, especially when considering the limited availability of trained professionals or specialists trained to identify ASD (5, 12, 13) and provide the required subsequent treatment (4). Furthermore, even when receiving treatment or intervention services such as speech therapy, occupational therapy, or behavior therapy, individuals are receiving services at a frequency below recommended therapeutic standards (14). Paula et al. (5) addressed barriers to care as reported by caregivers of autistic individuals in multiple Latin American countries, including Argentina. In said study, a high percentage of caregivers reported barriers to services reflected through long waiting lists, high treatment costs, and limited specialized services (5).

In addition to healthcare disparities and structural barriers to early ASD diagnosis and intervention, limited community awareness combined with unique cultural factors have been proposed to impact help-seeking behaviors (15). Latino parents within the United States report experiencing limited knowledge of ASD and resources available as well as cultural views that appear to be instrumental in delaying ASD diagnosis (16). Although there is evidence to suggest the experiences of parents in Latin American countries might be similar (5, 17) research exploring parent experiences regarding their understanding of ASD as well as information available to them and the community has been deficient (5, 18). This has begun to change considerably within the past decade, primarily through the self-advocacy movement and the establishment of parent support groups and autism associations. The establishment of organizations such as Red Espectro Autista (RedEA), which consists of representatives from various autism support groups, serves to increase ASD awareness, empower autistic individuals and family members, promote educational inclusion as well as political and social changes in Argentina (19, 20). In the area of research, networks such as Red Espectro Autista Latinoamericana (REAL) have been established to allow opportunities for Argentinian advocates and researchers to collaborate with other Latin American countries in research to promote increased awareness of ASD in Latin America (5). This movement toward advocacy and awareness has led to increased research opportunities focusing on interventions for parents (21, 22), as well as the validation of popular screening and diagnostic tools for Latin American populations (23–31).

Argentina has also passed laws, focusing on individuals with disabilities and in some cases focusing solely on ASD. For example, Act 27043 (the Comprehensive and Interdisciplinary Approach to persons with Autistic Spectrum Disorders) passed in 2014 with the aim to “ensure clinical and epidemiological investigation, early detection, diagnosis and treatment, dissemination and access to intervention services to autistic spectrum disorders” and to integrate early screening and diagnostic procedures along with required services into the Mandatory Medical Program (9). Yet, despite its passing, autistic Argentinians had to wait more than 5 years for this law to finally be legally implemented (32). This is unfortunate when one considers that early identification and diagnosis of ASD can lead to better outcomes for autistic individuals through the early access and utilization of intervention services (33). However, despite its benefits, for many autistic individuals, access to early intervention does not occur due to delayed diagnosis (17, 34). Although evidence indicates ASD can be diagnosed by 18 months of age, the average age of diagnosis for children in the United States is 4 years of age, and for some minorities, diagnosis occurs later (35–37). At present, there is limited information regarding the age of diagnosis for ASD in Latin America; however, some research estimates have identified a mean age of diagnosis at approximately 4.5 years of age with initial concerns having been noted at 2 years of age (14, 17, 38). This delay in diagnosis may profoundly impact the life trajectory of autistic individuals and their families.

Despite the limited understanding concerning the ASD experience for autistic individuals and their families in Argentina, this paper responds to the call made by the ASD community to represent better ASD knowledge outside the United States and European Countries (18) by exploring the changes in patterns of diagnosis and caregiver perceptions across a five-year span in Argentina. Also, it seeks to address the possible changes that have occurred since the previously mentioned non-profits launched and laws were implemented.

### Aims

The present study's aims are two-fold. First, quantitatively examine changes in the age of developmental concerns of autistic children, age of diagnosis, and diagnostic awareness across 5 years. For this purpose, we examined changes in the age and type of first concerns observed by caregivers, differences in age and type of diagnosis received by autistic children, and the association between the person who noticed the first concern (e.g., parents, physician or teacher) and the eventual age of diagnosis. The second aim is to identify changes in the challenges experienced by caregivers (e.g., reported service barriers) and the priorities and needs of Argentinian caregivers of autistic children (e.g., ensuring their child receives better services). This last aim would offer some information on the possible impact of implemented awareness campaigns and laws aimed at protecting individuals with disabilities on the reduction of systemic barriers in Argentina.

## Method

### Sample characteristics

Caregivers of autistic individuals completed an anonymous online questionnaire in Argentina at two different times, 2015 and 2020. Respondents were different each year the questionnaire was distributed. The 2015 survey consisted of 763 caregivers. Table 1 shows the demographic characteristics of both samples. For the 2015 sample, the majority of caregivers had some college or a higher educational degree (*n* = 534, 70.7%), and the most common diagnosis in their child was ASD or an autism diagnosis 34.1% (*n* = 260). In the 2020 sample, most of the sample had some college education or a higher degree as well (*n* = 341, 80.8%). Of the 422 respondents, the majority reported their child as autistic or ASD (*n* = 305, 72.3%). While children attending a private school was the top chosen alternative (*n* = 344, 49.4%) in the 2015 sample, in the 2020 sample, almost half of caregivers indicated the *other* alternative (*n* = 170, 47.9%). From this last group (2020), 31.5% (*n* = 54) of caregivers who endorsed the *other* category indicated that their child was attending a special school for children with disabilities. The age of autistic children was similar across samples, with most caregivers endorsing having a child older than 6 (Table 1). For both samples, inclusion criteria specified participants being at least 18 years old and the caregiver of an autistic child. The exclusion criteria were caregivers of individuals without an ASD reported diagnosis or those residing outside Argentina. To better allow for comparisons, both samples were drafted through similar channels. Both in 2015 and 2020, questionnaires were distributed through civil organizations like PANAACEA and RedEA, the largest autism non-profit agencies in Argentina. This allowed for samples to be comparable regarding autism awareness since participants in both were connected to similar organizations.

**Table 1:** Demographic characteristics of the sample.

	**2015**		**2020**		***x^2^***	***p***
	***N*** = **763**		***N*** = **422**		
	***N***	***%***	***N***	***%***		
**Caregiver education**					27.98	***
Primary	15	(2)	9	(2.1)		
Secondary	206	(27.3)	72	(17.1)		
Some college	257	(34)	130	(30.8)		
College degree	216	(28.6)	173	(41)		
Graduate degree	61	(8.1)	38	(9)		
**Age of child**					1.29	
Younger than 6	273	(35.8)	117	(39.5)		
Older than 6	490	(64.2)	179	(60.5)		
**Diagnosis**					182.101	***
Autism/ASD	260	(34.1)	305	(72.3)	
PDD-NOS	257	(33.7)	60	(14.2)	
PDD	128	(16.8)	47	(11.1)	
Asperger	118	(15.5)	10	(2.4)	
**Type of School**					37.13	***
Public school	276	(39.6)	78	(21.8)	
Private school	344	(49.4)	85	(23.8)	
Other	37	(5.3)	171	(47.9)	
No longer goes to school	40	(5.7)	23	(6.4)	
because of age		
**Available school assistance**	**355**	**(47.1)**	**145**	**(42.6)**	1.86	

*Includes valid responses only, may not total sample*.

*p < 0.05; **p < 0.01; ***p < 0.001.

### Procedure and instrument

Both surveys were adapted from the Caregivers Needs Survey (CNS) developed by Autism Speaks to better understand autistic individuals' needs (39). This is a retrospective study in which caregivers of autistic individuals were surveyed about their clinical and social history. The questionnaire inquired about: demographic information of caregivers and autistic individuals (e.g., age, educational attainment), first developmental concern (type and age it occurred), information on ASD diagnosis (e.g., age, provider who diagnosed, diagnostic label), service utilization, educational services, caregivers' perceptions, perceived impact of ASD, stigma, quality of life, and challenges and priorities. For both adapted versions of the survey, the CNS Spanish translation was reviewed for appropriateness and adapted by REAL clinicians who work with children with developmental disorders and their families. Following this adaptation, caregivers of autistic individuals examined the instrument, and wording was modified better to reflect autistic individuals and their families' experiences. The final version of each survey differs in some items, yet similar items across both samples were compared for this study's purpose. These comparisons were on items regarding demographic characteristics of autistic children and caregivers, service barriers, diagnostic and first concern information, and caregivers' perceptions regarding rights and needs. Montenegro et al. (40), Montiel-Nava et al. (14), and Paula et al. (5) describe the adaptation process for the 2015-version of the survey. This is the first publication of the 2020 version. Both versions were distributed online. After entering the website and prior to start completing the questionnaire, participants needed to provide their consent by typing their initials. Approval to conduct this study was obtained through a local ethics board.

### Statistical analysis

Data was filled out in excel files and merged in SPSS for the statistical analysis. Comparisons between items in the two surveys were conducted through descriptive inferential analysis and chi-square tests of independence At least 80% of expected cell frequencies were greater than five. All statistical analyses were performed using IBM SPSS, version 26.

### Variables

#### Respondents characteristics

Both surveys inquired about demographic information by asking about caregiver education (categorical), child diagnosis (categorical), and the type of schooling the child attended (categorical). In addition, the samples included autistic adults that could have been diagnosed while the PDD category was the current one.

#### First concerns

To better understand caregivers concerns regarding their child's development, type of first observed concern and the age at which it occurred were queried. For the observed first concern, caregivers could choose among six types (categorical; medical problems, behavioral difficulties, no response to sounds or names, insistence on sameness or difficulty with changes, and gross motor problems). Caregivers could pick one or multiple concerns that applied to their child. Age of first observed concern was categorized in ranges (0–12 months, 12–18 months, 18–24 months, 24–36 months, 36–72 months and 72 months and up). For the person noticing first concern, alternatives to choose from were family members, doctor, and teacher (categorical, see Tables 2, 3).

**Table 2:** First developmental concern and age of diagnosis.

	**2015** ***N*** = **763**		**2020** ***N*** = **422**		***x^2^***	***p***
**Age of first concern**					113.27	***
0–12 months	108	(14.4)	82	(19.6)		
12–18 months	85	(11.4)	121	(28.9)		
18–24 months	142	(19.0)	101	(24.2)		
24-36 months	249	(33.3)	79	(18.9)		
36–72 months	155	(20.3)	30	(7.2)		
72+ months	9	(1.2)	4	(1.0)		
Observed first concern*						
Medical problems	135	(19)	64	(15.2)	2.64	
Behavior difficulties	460	(61.8)	117	(27.7)	125.28	***
No response to	473	(63.9)	267	(63.3)	0.37
sounds/name			
Sameness/difficulty	466	(63)	179	(42.4)	45.98	***
with changes				
Gross motor problems	178	(24.5)	97	(23)	0.329	
**Age of diagnosis**					15.136	**
0–3 years old	500	(66.4)	325	(77.2)	
4–8 years old	201	(26.7)	77	(18.3)	
9–12 years old	34	(4.5)	12	(2.9)	
13–17 years old	13	(1.7)	2	(0.5)		
18+ years old	5	(0.7)	5	(1.2)		

*Includes valid responses only, may not total sample*.

*p < 0.05; **p < 0.01; ***p < 0.001.

**Table 3:** Sample year and individual who noticed first developmental concern.

	**2015** ***N*** = **747**		**2020** ***N*** = **392**		***x^2^***	***p***
	***N***	***%***	***N***	***%***		
**First person noticed concern**					47.32	***
Family member	612	(81.9)	253	(64.5)		
Physician	54	(7.2)	72	(18.6)		
Teacher	81	(10.8)	130	(16.8)		

*Includes valid responses only, may not total sample*.

*p < 0.05; **p < 0.01; ***p < 0.001.

#### Service barriers and challenges

Multiple alternatives were provided for types of barriers when accessing services for their autistic child (e.g., not qualifying for services, services not available in the area, among others, **Table 5**). Caregivers could indicate more than one service barrier (binary, yes and no). To assess financial impact of ASD, participants were asked whether a family member ceased work to care for autistic child (binary, yes and no). Binary options were provided concerning challenging characteristics observed in autistic children, and caregivers could endorse more than one (e.g., problematic behaviors, daily living skills, health problems, and so on). These challenging behaviors were summed together to assess number endorsed by caregivers (**Table 6**).

#### Age of diagnosis

Age of diagnosis was categorized into ranges (0–3, 4–8, 9–12, 13–17, and 18+ years old) (Table 4).

**Table 4:** Age of diagnosis and person who first noticed developmental concern.

	**Family member**		**Physician**		**Teacher**		**Total**			
**Age of diagnosis**	***N***	***%***	***N***	***%***	***N***	***%***	***N***	***%***	***x^2^***	***p***
**2015**									39.95	***
0–3 years	427	(86.6)	37	(7.5)	29	(5.9)	493	(100)		
4–8 years	140	(71.4)	15	(7.6)	41	(21)	196	(100)		
9 years and older	40	(87)	2	(4.3)	4	(8.7)	46	(100)		
**2020**									45.09	***
0–3 years	215	(71.4)	57	(19)	29	(9.6)	301	(100)		
4–8 years	33	(37.5)	14	(16)	41	(46.5)	88	(100)		
9 years and older	5	(33.3)	1	(6.7)	9	(60)	15	(100)		

*Includes valid responses only, may not total sample*.

*p > 0.05; **p < 0.01; ***p < 0.001.

#### Priorities

Caregivers' perceived priorities in terms of support, community awareness, and autistic individuals' rights were measured using binary alternatives (yes and no). Caregivers could choose more than one item (Table 5).

**Table 5:** Differences between family member ceasing work and challenging behaviors.

	**Ceased work**		***x^2^***	***p***
	**(*****N*** = **1,176)**			
Number of challenging behaviors			22.14	***
0–1	25	(6.2)		
2–3	336	(83.6)		
More than 4	41	(10.2)		

*Includes valid responses only, may not total sample*.

*p < 0.05; **p < 0.01; ***p < 0.001.

## Results

### Age of first concerns

In terms of the age of first concerns, there were significant differences between the two samples (*x*^2^*(9)* = 113.274, *p* < 0.001). Caregivers noticed developmental first concerns earlier in the 2020 sample. The category of concern noticed at 0–12 months increased from 14.4 % (*n* = 108) in 2015 to 19.6% (*n* = 82) in 2020. This also occurred with the age range of 12–18 months with 11.4% (*n* = 85) in 2015 and 28.9% (*n* = 121) in 2020, and 18–24 months which increased from 19% (*n* = 142) in 2015 to 24.2% (*n* = 101) in 2020. Overall, in the 2015 sample, 44.8% (*n* = 335) of caregivers noticed a first developmental concern before their child's 24 months, whereas 72.7% (*n* = 304) did so in the 2020 one.

### Type of first concerns

There was no statistically significant difference (*x*^2^*(1)* = 0.37, *p* > 0.05) when comparing the most common observed first concern, *not responding to name*, which remained the highest choice with ~64% of caregivers in both samples endorsing it. Both samples' least common first concern was the presence of medical problems (*x*^2^*(1)* = 2.34, *p* > 0.05), remaining below 20% (Table 2).

Differences across samples were identified (*p* < 0.001) for behavior difficulties (*x*^2^*(1)* = 125.28) and insistence on sameness (*x*^2^*(1)* = 45.98). Frequency of caregiver reporting of behavior difficulties decreased from 61.8% (*n* = 460) in the 2015 sample to 27.7% (*n* = 117) in 2020, whereas insistence on sameness decreased from 63 (*n* = 466) to 42.4% (*n* = 179).

### Age of diagnosis and type of diagnosis

When comparing age of diagnosis (*x*^2^*(2)* = 15.14, *p* = 0.002) and type of diagnosis *(x*^2^*(4)* = 182.10, *p* < 0.001) there was a statistical significance differences between both samples. For the 2015 sample, 34.1% of caregivers indicated their child having an ASD or autism diagnosis. This was closely followed by PDD-NOS diagnosis (*n* = 257, 33.7%). For age of diagnosis, most caregivers (*n* = 500, 66.4%) indicated their child was diagnosed before the age of three. Yet almost 30% (*n* = 201) were diagnosed between 4 and 8 years old. On the other hand, in the 2020 sample, autism or ASD diagnosis increased to 77.3% (*n* = 305), whereas PDD-NOS decreased to 14.2% (*n* = 60). Diagnosis before the age of 3 years old rose to 77.2% (*n* = 325), and diagnosis between ages 4 and 8 decreased to 18.3% (*n* = 77).

### Age of diagnosis and individual who noticed first concern

There was a statistically significant difference (*x*^2^*(2)* = 47.32, *p* < 0.05) between individual (family member, physician, teacher) who noticed first concerns about child's development and sample year. As exhibited in Table 3, family members are the most frequent individuals who notice developmental concerns in both samples. In contrast, teachers had second place in the 2015 sample (10.7%, *n* = 81), while physicians had it for the 2020 sample (18.6%, *n* = 16.8). A chi-square test of independence was also conducted to assess frequency differences in individual who noticed first concern and the age of diagnosis of the autistic child in each sample year. This analysis showed statistically significant differences between the person who noticed the first concern and the age of diagnosis for both the 2015 sample (*x*^2^*(6)* = 39.95, *p* < 0.001) and 2020 one (*x*^2^*(6)* = 45.09, *p* < 0.001). For the 2015 survey, almost 90% (*n* = 427) of participants who indicated a family member noticing first developmental concern had their child diagnosed on or before 3 years of age. Among those diagnosed before the age of three, only 7.5% had their first concern noticed by a physician (*n* = 37), and 5.9% by a teacher (*n* = 29). In the 2020 sample, 19% (*n* = 57) had a physician and 9.6% a teacher (*n* = 57) noticing the first developmental concern. Among those children diagnosed between the ages of 4 and 8, in the 2015 sample, 21% of teachers noticed first concern (*n* = 41), while in the 2020 sample, 46.5% had their teacher noticing (*n* = 215); showing an increase of teacher's awareness.

### Service barriers and caregivers challenges

Caregiver reports of service barriers decreased in all categories. Experienced delays due to waitlist significantly decreased from 43.5% in 2015 to 16.4% in 2020 (*x*^2^*(1)* = 81.97, *p* < 0.001), treatment cost from 33.5% to 12.6% (*x*^2^*(1)* = 60.99, *p* < 0.001), limited information from 17.9 to 4.3% (*x*^2^*(1)* = 43.98, *p* < 0.001), not qualifying for services from 18.2 to 9.2% (*x*^2^*(1)* = 16.76, *p* < 0.001), and service not available in their area decreased from 26.5 to 18% (*x*^2^*(1)* = 10.78, *p* = 0.001). When exploring the financial impact of caring for an autistic individual, caregivers' reports of a family member having to stop working to care for their child showed no statistical significance (*p* > 0.05) when comparing both samples, with more than 34% of caregivers indicating agreement (*x*^2^*(2)* = 0.57, *p* > 0.05). There was a statistically significant difference between the amount of challenging behaviors and the number of those reporting that a family member had ceased working (*x*^2^*(2)* = 22.14, *p* < 0.05), showing an increased rate of family member ceasing work as number of challenging behaviors increased (Table 5).

Concerning autistic characteristics that were challenging for caregivers, there were no statistically significant differences in social interaction difficulties which showed the highest rate in both samples (*x*^2^*(1)* = 0.12, *p* > 0.05) with an almost 55% endorsement. Differences were identified, with an increased number of participants from the 2020 sample reporting greater concerns in several areas. Communication difficulties were endorsed at a significantly higher rate (*x*^2^*(1)* = 14.18, *p* < 0.001) in 2020 (55%) than in 2015 (43.5%). Moreover, sensory issues (*x*^2^*(1)* = 68.65, *p* < 0.001) saw an increase with 11.5% in 2015 vs. 31% in 2020, restricted and repetitive behaviors (*x*^2^*(1)* = 15.68, *p* < 0.001) with 20.3% in 2015 and 30.6% in 2020, sleep problems (*x*^2^*(1)* = 34.67, *p* < 0.001) with more than double the rate in 2020 (22.5%), and eating or feeding difficulties also with little more than double the percentage of caregivers within the sample (28.9%) indicating endorsement in 2020. Significant decreases in challenges were also identified with impaired sense of safety and notion of danger (*x*^2^*(1)* = 8.54, *p* = 0.003) decreased from 20.6 to 13.7% (Table 6).

**Table 6:** Service barriers, challenges, and priorities.

	**2015** ***N*** = **763**		**2020** ***N*** = **422**		***x^2^***	***p***
**Service barriers**						
Not qualifying for services	131	(18.2)	39	(9.2)	16.76	***
Not available in their area	191	(26.5)	76	(18)	10.78	**0.001
Waiting list	245	(43.5)	69	(16.4)	81.97	***
Treatment costs	243	(33.5)	53	(12.6)	60.99	***
Limited Information	127	(17.9)	18	(4.3)	43.98	***
Other	122	(17.8)	57	(13.5)	3.5	***
**Family member ceased working**	257	(34.1)	145	(34.3)	0.57	
**Challenges**					
Challenging behaviors	276	(36.2)	133	(31.5)	2.61	
Daily living skills difficulties	260	(34.1)	166	(39.3)	3.23	
Health problems	48	(6.3)	23	(5.5)	0.34	
Sleep problems	76	(10)	95	(22.5)	34.67	***
Eating/feeding difficulties	109	(14.3)	122	(28.9)	37.03	***
Social interaction difficulties	413	(54.1)	224	(53.1)	0.12	
Restricted/repetitive behaviors	155	(20.3)	129	(30.6)	15.68	***
Communication difficulties	332	(43.5)	232	(55)	14.18	***
Impaired safety/notion of danger	157	(20.6)	58	(13.7)	8.54	0.003
Sensory	88	(11.5)	131	(31)	68.65	***
**Priorities**					
Receiving social support	285	(37.4)	105	(24.9)	19.14	***
Basic rights protected	480	(19.0)	266	(18.7)	0.02	
Better health services	363	(47.6)	113	(26.8)	48.91	***
Better education services	444	(58.2)	222	(52.6)	3.44	
More rights for autistic individuals	198	(26)	132	(31.3)	3.84	*
Improved implementation existing rights	169	(22.1)	187	(44.3)	63.51	***
More information about ASD	176	(23.1)	137	(32.5)	12.35	***
Home support	97	(12.7)	68	(16.1)	2.62	
Community awareness	310	(40.6)	194	(46)	3.17	

*Includes valid responses only, may not total sample. Percentages are within sample year. Since caregivers could choose more than one option percentage total does not equal 100%*.

*p < 0.05; **p < 0.01; ***p < 0.001.

### Caregiver's priorities

Priority being placed on better educational services continued to be the most frequently chosen item among caregivers, with more than 50% doing so; however, there was no statistically significant difference (*x*^2^*(1)* = 3.44, *p* = 0.003) between the two groups. Significant differences among both samples were for caregivers wanting: more rights for autistic individuals (*x*^2^*(1)* = 3.84, *p* = 0.5), improved implementation of existing rights (*x*^2^*(1)* = 63.51, *p* < 0.001), and more information about ASD (*x*^2^*(1)* = 12.35, *p* < 0.001). All of these had higher endorsement by caregivers in the 2020 sample (see Table 2). Whereas, rates of priorities being that autistic child receives social support (*x*^2^*(1)* = 19.14, *p* < 0.001) and better health services (*x*^2^*(1)* = 48.91, *p* < 0.001) decreased in the 2020 sample. On the contrary, basic rights being protected (*x*^2^*(1)* = 0.02, *p* > 0.05), home support (*x*^2^*(1)* = 2.62, *p* > 0.05), and community awareness (*x*^2^*(1)* = 3.17, *p* > 0.05) showed no statistically significant difference between the two samples.

## Discussion

Our first goal was to observe quantitative differences in the age of first concerns, individuals who first noticed developmental concerns, and changes in the age of diagnosis. In our latest sample, more caregivers reported first concerns before their child was 24 months. In line with research from other countries (41), our results showed an increased caregivers' awareness in the 2020 sample, as evidenced by more caregivers reporting their child's first developmental concern before the age of 24 months. In addition, it highlights the importance of caregivers as an essential element for identifying ASD early signs (37). In our study, caregivers were the most likely to notice early concerns instead of physicians and teachers. This rate increase of earlier diagnosis noticed by caregivers may reflect a better understanding of ASD in Argentina which has been promoted by advocacy groups that have advocated for the enforcement of policies, laws, and adherence to the Convention of the Rights of Persons with Disabilities. This convention, which Argentina and other countries adhered to, promotes attitude changes to improve the quality of life of disabled individuals and decrease barriers toward the inclusion and protection of those with disabilities (14). Previous literature has indicated how better awareness could help with earlier recognition and stigma reduction (42). It is thus imperative to continue advocacy work and the implementation of strategies that promote increased community awareness and global health response toward an increased community capacity (1). This is particularly relevant when considering that a lack of ASD knowledge is associated with misconceptions and a deficit view of the condition, which conceptualizes ASD as an illness (43). Additionally, an increased attention and appropriate information could permit improved accessibility to better quality intervention and treatment services (34) and alleviation of symptom severity (33).

Regarding types of first observed concerns, lack of response to name and insistence on sameness remained consistently high in the latest sample. These two characteristics are part of the diagnostic criteria identified in the DSM-5 and ICD-10 (44, 45). Not responding to their name is encompassed in deficits in social-emotional reciprocity (44). On the other hand, caregivers from the 2020 sample were least likely to endorse behavior difficulties and medical and gross motor concerns as first developmental concerns. This could be explained by ASD diagnostic criteria since, despite aggression, medical conditions, and gross motor delays being commonly co-occurring conditions (46), they are not required to meet an ASD diagnosis (47, 48). Our results seem to imply an attunement in identifying ASD core characteristics. However, more than 20% of caregivers continue to report common co-occurring conditions as the first noticed developmental concerns. These results highlight the need for service providers and clinicians to hear caregivers' reports of co-occurring conditions due to their increased prevalence among autistic individuals compared to typically developing children (48). Also, as previously indicated, caregivers continue to notice developmental concerns earlier.

In both samples, of those children diagnosed before the age of three, most caregivers (>70%) had noticed the first developmental concern. In other words, when caregivers noticed a developmental difference in their children, those children were more likely to be diagnosed earlier. Yet, interestingly, in the 2020 sample, the frequency of children diagnosed before the age of three who had a physician or teacher noticing the first developmental concern increased. These results imply two things: caregivers seem to be heard by providers who diagnose their children before preschool age, and physicians and teachers seem to have more awareness and knowledge. This possible increased awareness might reflect the proactive work being conducted in Argentina, which has taken an active and continuous stance in promoting awareness and inclusion of those individuals with disabilities. For instance, non-profit organizations, such as RedEA, work toward greater community awareness and advocacy for quality-of-life improvement (19, 20, 49). Moreover, Argentina has established laws that protect individuals with disabilities, particularly autism. In 2014 the National Autism Law (previously mentioned as Act 27043) passed and was implemented legally by 2019. This law is complemented by the National Disability Law (20). These laws mandate better accessibility to diagnosis and healthcare and emphasize a comprehensive and interdisciplinary approach, the training of healthcare professionals, and increased research efforts (20). Therefore, changes in decreased age of first concerns, age of diagnosis, and the individual who noticed developmental differences might reflect changes in community awareness and increased knowledge brought upon by the work of non-profits, advocates, and the implementation of policies which focus on the protection of those with ASD. This information is relevant to other countries, which might still be behind in disability laws and advocacy work. For example, in Latin America, Argentina is the only country that has a legal framework focusing on those with ASD (14).

Our second goal was to identify challenges experienced by caregivers and address possible gaps in policies and practices concerning services, rights, and support. In the 2020 sample, fewer parents indicated accessibility issues when observing service barriers. Despite these results pointing to a positive trend in service provision in Argentina, a large percentage of families reported still facing barriers. One of those challenges that remained steadfast across both samples was family members continue quitting their work to care for an autistic individual. This is problematic when considering the high cost of caring for somebody with ASD (50). For families already struggling with financial challenges, eliminating one source of income could translate to an even more distressing situation, particularly for those whose children have more severe symptomatology (51, 52). Horlin et al. (51) indicated that 90% of the family cost related to ASD is due to loss of income. Additionally, increased cost was associated with the number of ASD symptoms (51). In our sample, reports of challenging characteristics such as social interaction, sensory issues, and restrictive, repetitive behaviors remained high or increased among caregivers. Also, in both samples, most caregivers reported their child having more than two challenging behaviors, among which more than 90% had a family member ceasing to work to care for their autistic child. It is thus expected that for these caregivers, the cost of caring for an autistic individual means added financial impact. The added cost of ASD and the loss of income for Argentinian caregivers could be exceptionally burdensome given the precarious economic affairs currently plaguing the country. From 2015 to 2020, many of the country's financial indicators have shown a negative trend, with its gross domestic product (GDP) decreasing by more than 200 billion dollars (7). To understand how significant this sum is, one can compare it to Argentina's current GDP of 389 billion (7). In 2015 1 in every 3 Argentinians was living below the poverty line, and this economic situation did not improve in the following years, resulting in austerity measures and a long-lasting recession lasting until this day (53). In our sample, the added number of problematic behaviors combined with family members ceasing to work in an environment struggling with recession and poverty implies more challenges requiring caregivers of autistic individuals to reorganize their priorities to meet their child's needs better (54).

Although there have been similarities and differences observed in the challenges expressed by caregivers, caregiver priorities have remained relatively consistent across both samples. Child education and receiving better educational services remained the highest endorsed priority. The second most endorsed priority was to improve the implementation of existing rights (increased endorsement in the 2020 sample) and increased community awareness (similar rates in both samples). Reporting of educational services as a continued priority is understandable. ASD cases in the mainstream school system have seen a 25-fold increase in recent years (55). The rise in cases meant an increased need for more school personnel that understands developmental concerns, and this need translated to the use of support teachers that form part of the specialized support in schools (55). Also, despite Argentina establishing laws outlining policies for the educational inclusion of children with ASD, how these policies and laws are operationalized has yet to be determined. Therefore, there is little accountability when it comes to enforcing such legislature, and concerns regarding the quality of education for ASD children have been raised (55). For example, teacher training programs do not provide up-to-date ASD information in curricular programs which could result in teachers not being adequately trained to implement inclusive practices in school settings (55). Based on our identified results, it is imperative to have more efficient teachers and support staff training on ASD and, include a more precise operationalization on the needs of an inclusive school system.

Although Argentina has a legal framework aiming to to protect the rights of those with ASD, there are certain limitations to its implementation. For example, some of those laws refer to disabled people as “people that suffer” from said disability, promoting stereotypes and prejudice. Such language across legal documents perpetuates the categorization of autistic individuals as handicapped and thus silencing the heterogeneity and neurodiversity within the spectrum (56). In addition, complex bureaucracy, extensive and unclear paperwork limit laws implementation. Argentina disability laws clearly outline policies to establish the rights of children with ASD, including educational inclusion and feasible access to appropriate diagnosis and treatment; however, there is not a mechanism explaining how these policies are operationalized (56). Currently, caregivers of autistic individuals and self-advocates have organized several activities to protest for which they consider a failure to comply with legally established laws resulting in multiple children not receiving an ASD diagnosis, which in turn delays access to timely treatment (57). Despite laws aiming at protecting the rights of those with disabilities in Argentina, many families do not know how to navigate a somewhat cumbersome system. For example, in Argentina, the previously mentioned *Certificado Único De Discapacidad* (Unique Certificate of Disability, UCD) is a public document that enables individuals with disabilities to exercise their rights and access social benefits as described by national laws (58). Yet only 14.6% of individuals with disabilities have access to the UCD (9). Families of individuals with disabilities confront an uncharted territory when trying to find diagnosis and treatment while navigating “bureaucratic obstacles, originating from its health system and society” [(9), p. 355]. These obstacles in addition to the limited knowledge of existing rights, increases worry, uncertainty, and exhaustion in families, which further hinders autistic individuals' full inclusion in society (9).

Community awareness continues to be reported by caregivers as a priority. It is important to mention that Argentina has made strides toward increased community awareness. As previously mentioned, non-profit organizations' main goal is to increase awareness and empower autistic individuals. Additionally, in 2015, an important ASD awareness campaign titled *Mirame* won the first prize for a recognized national competition (59). This campaign had a significant public impact and increased its website visitors, which included information on autism screening, early signs recognition, and available local services (5). Due to the continued endorsement of community awareness as a priority, future research could further explore caregiver input on increasing awareness.

Our study provides a window into the lives of Argentinian caregivers. Its ability to assess their experiences within a 5-year gap also allows for the observation of differences across time on multiple aspects such as first developmental concerns, age of diagnosis, challenges and priorities of caregivers of autistic individuals. In Argentina, GDP expenditure dedicated to research is only 0.49%, whereas in countries like the United States estimated expenditure is at 2.20% (7). Elsabbagh et al. (18) indicated that health research funding in multiple countries is limited with only 10% of the global healthcare expenditure going to 90% of the world's population (18). Yet, through the present study, researchers and specialists in Argentina have responded to the call made by previous publications to fill the ASD knowledge gap in other parts of the world outside the United States and Europe (18, 60).

Despite its contributions, the present study presents some limitations such as the lack of confirmatory diagnosis of the children in the study. Also, most respondents in both samples had some form of higher education and thus did not represent those with lower educational attainment. Lastly, there is limited background information to further make comparisons between both populations from which the two samples were drawn. As such, despite both samples being derived from similar channels to enhance similarities, one cannot discard baseline differences which could be contributing to the results presented. Yet, knowledge gathered from this study could help elucidate possible progress in Argentina in terms of age of diagnosis, caregiver increased awareness of developmental concerns, and type of concerns caregivers continue to notice in their autistic children. Through caregivers' challenges and perceived priorities, policy and lawmakers can gain insight into the work still needed to be done for better educational inclusion and implementation of protective rights.

Taken together, this study's results imply an improvement in the notice of developmental concerns, a decreased age of diagnosis, and an improvement in several services in caregivers of autistic individuals in Argentina. Nonetheless, many caregivers reported barriers or rights still needing to be protected or improved. Our findings help illustrate not only Argentina's reality in terms of their ASD experience but also help inform of the possible steps toward greater community engagement and implementation of changes in public policies and practice in other Latin American countries.

## Data availability statement

The raw data supporting the conclusions of this article will be made available by the authors, without undue reservation upon request.

## Ethics statement

The studies involving human participants were reviewed and approved by Universidad Catolica Argentina (UCA). The patients/participants provided their written informed consent to participate in this study.

## Author contributions

MM, EB, SC, DV, and CM-N contributed to conception and design of the study. SC, DV, and ARa collected the data. MM organized the database and performed the statistical analysis. MM and EB wrote the first draft of the manuscript. CM-N wrote sections of the manuscript. All authors contributed to manuscript revision, read, and approved the submitted version.

## Conflict of interest

The authors declare that the research was conducted in the absence of any commercial or financial relationships that could be construed as a potential conflict of interest.

## Publisher's note

All claims expressed in this article are solely those of the authors and do not necessarily represent those of their affiliated organizations, or those of the publisher, the editors and the reviewers. Any product that may be evaluated in this article, or claim that may be made by its manufacturer, is not guaranteed or endorsed by the publisher.
